# Place-Based Attributes Predict Community Membership in a Mobile Phone Communication Network

**DOI:** 10.1371/journal.pone.0056057

**Published:** 2013-02-22

**Authors:** T. Trevor Caughlin, Nick Ruktanonchai, Miguel A. Acevedo, Kenneth K. Lopiano, Olivia Prosper, Nathan Eagle, Andrew J. Tatem

**Affiliations:** 1 Department of Biology, University of Florida, Gainesville, Florida, United States of America; 2 School of Natural Resources and Conservation- Department of Wildlife Ecology and Conservation, University of Florida, Gainesville, Florida, United States of America; 3 Department of Statistics, University of Florida, Gainesville, Florida, United States of America; 4 Department of Mathematics, University of Florida, Gainesville, Florida, United States of America; 5 MIT Media Lab, Cambridge, Massachusetts, United States of America; 6 Santa Fe Institute, Santa Fe, New Mexico, United States of America; 7 Department of Geography, University of Florida, Gainesville, Florida, United States of America; 8 Emerging Pathogens Institute, University of Florida, Gainesville, Florida, United States of America; 9 Fogarty International Center, National Institutes of Health, Bethesda, Maryland, United States of America; Universidad Carlos III de Madrid, Spain

## Abstract

Social networks can be organized into communities of closely connected nodes, a property known as modularity. Because diseases, information, and behaviors spread faster within communities than between communities, understanding modularity has broad implications for public policy, epidemiology and the social sciences. Explanations for community formation in social networks often incorporate the attributes of individual people, such as gender, ethnicity or shared activities. High modularity is also a property of large-scale social networks, where each node represents a population of individuals at a location, such as call flow between mobile phone towers. However, whether or not place-based attributes, including land cover and economic activity, can predict community membership for network nodes in large-scale networks remains unknown. We describe the pattern of modularity in a mobile phone communication network in the Dominican Republic, and use a linear discriminant analysis (LDA) to determine whether geographic context can explain community membership. Our results demonstrate that place-based attributes, including sugar cane production, urbanization, distance to the nearest airport, and wealth, correctly predicted community membership for over 70% of mobile phone towers. We observed a strongly positive correlation (r = 0.97) between the modularity score and the predictive ability of the LDA, suggesting that place-based attributes can accurately represent the processes driving modularity. In the absence of social network data, the methods we present can be used to predict community membership over large scales using solely place-based attributes.

## Introduction

Social networks can be used to model many types of interactions between people, including friendship [1], disease transmission [2], and sexual contact [3]. Because network analysis allows key properties of otherwise complex systems to be represented by simple metrics, the study of social networks has revolutionized our understanding of a range of fields, including behavioral psychology [1], public health [3]–[5], and regional conflict [6]. One key property of social networks is modularity, the degree to which the network can be partitioned into communities of nodes with a relatively higher density of connections within the same community than between communities [7]. Modularity structure results in higher rates of disease spread [2], criminal activity [8], and movement [9], between nodes located in the same community. Consequently, understanding the processes that determine modularity in social networks is an important research goal.

For networks in which nodes represent individual people, general principles explaining community formation incorporate individual attributes. Homophily, the principle that similar individuals are more likely to interact, results in communities of individuals with similar attributes, such as ethnicity, gender, income, political views and more [10], [11]. Focus constraints, including shared activities, such as attending the same class at a university, may also lead to community formation [12]–[13]. Finally, spatial proximity, regardless of other shared activities or social attributes, may promote communities in social networks [14]–[15]. A challenge remains in translating these general principles for community formation in networks of individual people to large-scale social networks, in which network nodes represent a population of people at a given location. Examples of edges and nodes in these large-scale social networks include human movement between regions, patient transfer between hospitals, and criminal offenses between census tracts [4], [8]–[9].

Quantifying the importance of principles like homophily or focus constraints for network structure requires data on attributes of each node in the network. In large-scale networks, defined by a population at a location, attributes are place-based, representing the economic, social and ecological characteristics that define the geographic context of a location. While high modularity has been described for several large-scale social networks [6]–[8], whether or not place-based attributes can explain community membership in these large-scale social networks remains unknown. Aggregating data on individual attributes to create place-based attributes could be problematic if the mixture of individuals at a given location is too heterogeneous to represent as a single group. Another major challenge in using place-based attributes to explain patterns of connectivity in large-scale social networks is that space could overwhelm the effects of place-based attributes [15]. Resolving these uncertainties will require quantitative models to test whether various place-based attributes can explain module structure in large-scale social networks.

High modularity has been observed in large-scale social networks constructed from mobile phone communication between cell phone towers [6], [14]. Because mobile phone communication is correlated with friendship networks [1] and human movement [16]–[17], the ability to predict patterns, including modularity, in communication networks would have many useful applications in a great variety of fields. For example, mobile phone communication data could be used in epidemiology to model human movement between patches with high and low disease transmission risk [18]–[19]. However, mobile phone towers are absent for many locations within countries or regions. Consequently, extrapolating communities formed by call flow connectivity across an entire region requires predicting community membership for areas without mobile phone towers. If geographic context of mobile phone towers can explain modularity, quantifying place-based attributes may enable regional predictions of community membership in areas with and without mobile phone towers.

Here, we quantified whether place-based attributes can predict community membership for a large-scale social network of communication between mobile phone towers in the Dominican Republic. While previous studies have shown that individual attributes can predict community membership in person-to-person social networks, our study goes further to ask whether place-based attributes can predict communities in this large-scale tower-to-tower social network. We assigned towers to communities using a modularity algorithm then applied a linear discriminant analysis (LDA) to evaluate whether a set of four place-based attributes, including urbanization, area used for sugar cane production, distance to nearest airport, and wealth, could correctly predict tower community membership.

## Results

**Figure 1:**
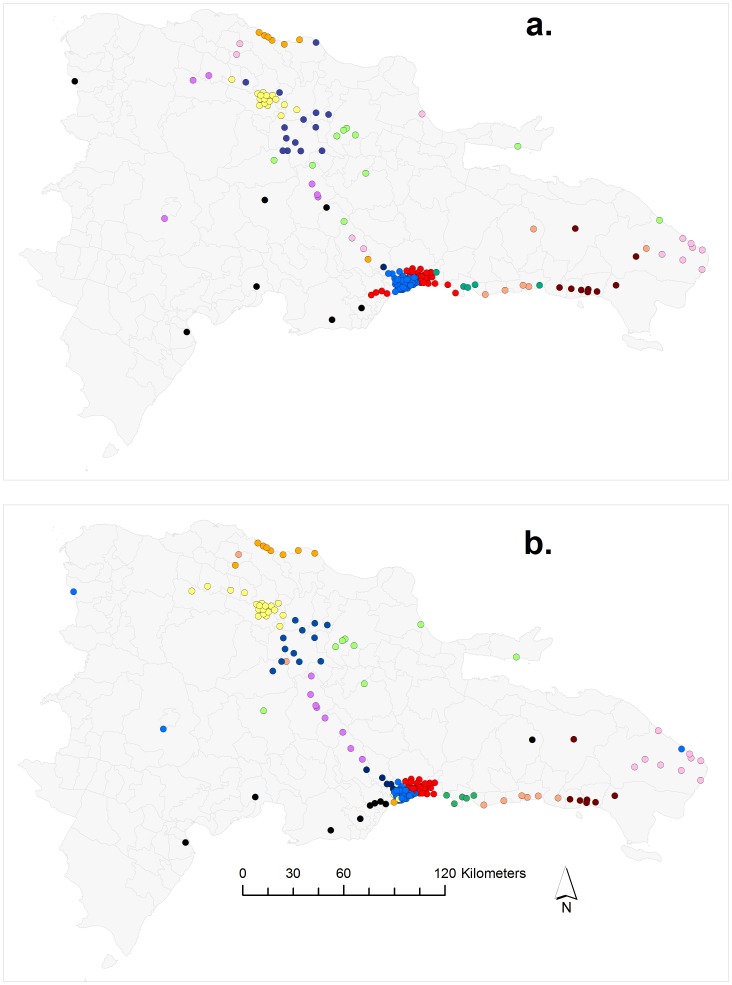
Community membership of 170 mobile phone towers in the Dominican Republic. Each dot represents a mobile phone tower, and each color represents a unique community. The capital city, Santo Domingo, is located in the center of the southern coast, represented by the cluster of red and blue dots. Panel a (top) shows the result from the run of the modularity algorithm with the highest modularity score. Panel b (bottom) shows predicted community membership of mobile phone towers from the LDA using four place-based attributes for the modularity realization with the top modularity score.

**Figure 2:**
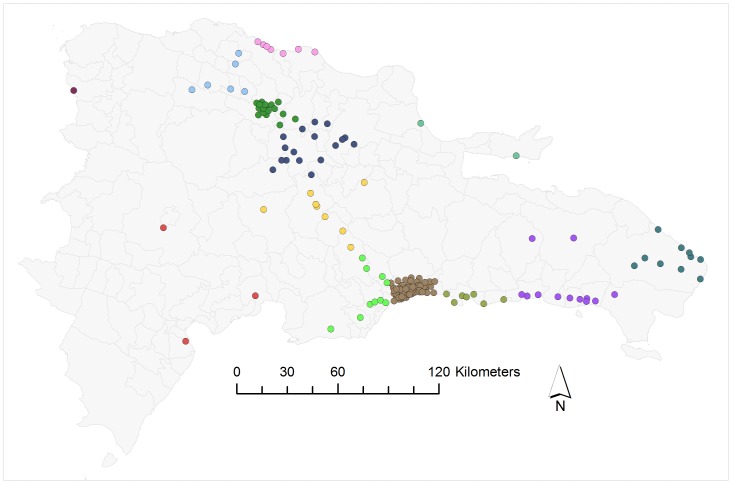
Predicted communities from k-means clustering, based upon the spatial location of each tower (here, k = 12). Each mobile phone tower is represented by a circle. The color of the circle indicates community membership.

First, we determined community membership for each tower in the network (Figure 1). For 100 separate runs of the modularity algorithm used to detect communities within the DR communication network, the modularity score ranged from a minimum of 0.26 to a maximum of 0.52. The results for community membership of mobile phone towers from the run with the highest modularity (hereafter “top” run) can be seen in Figure 1. The modularity algorithm maximizes the modularity score based on both community membership and total number of communities in the data, and the community structure that maximized this modularity score contained 13 different communities. Results from the top 20 runs of the modularity algorithm suggest that the simulated annealing algorithm had reached a plateau of modularity scores, with very similar output for these top runs. The mean (±SD) of modularity score for the top 20 runs of the modularity algorithm was 0.52±0.002, and the mean number of communities generated by these top 20 runs was 10.9±1.65. The core communities in these top 20 runs were also qualitatively similar (Figure S1). Notable features of the community structure include the division of the capital city of Santo Domingo into two separate communities (represented by red and blue dots in Figure 1), as well as the close links between towers on the border between Haiti and the Dominican Republic with the western half of the capital city (blue dots). The 13 described communities from the top modularity run appear clustered in space, however a clustering algorithm based only on node locations produced a simpler geographic pattern (Figure 2) than revealed by the cell phone data (Figure 1a). Therefore, to better explain the call patterns and community memberships, we used a linear discriminant analysis (LDA) to relate community membership to the four place-based attributes (sugar cane production, distance to nearest airport, urbanization and wealth).

**Figure 3:**
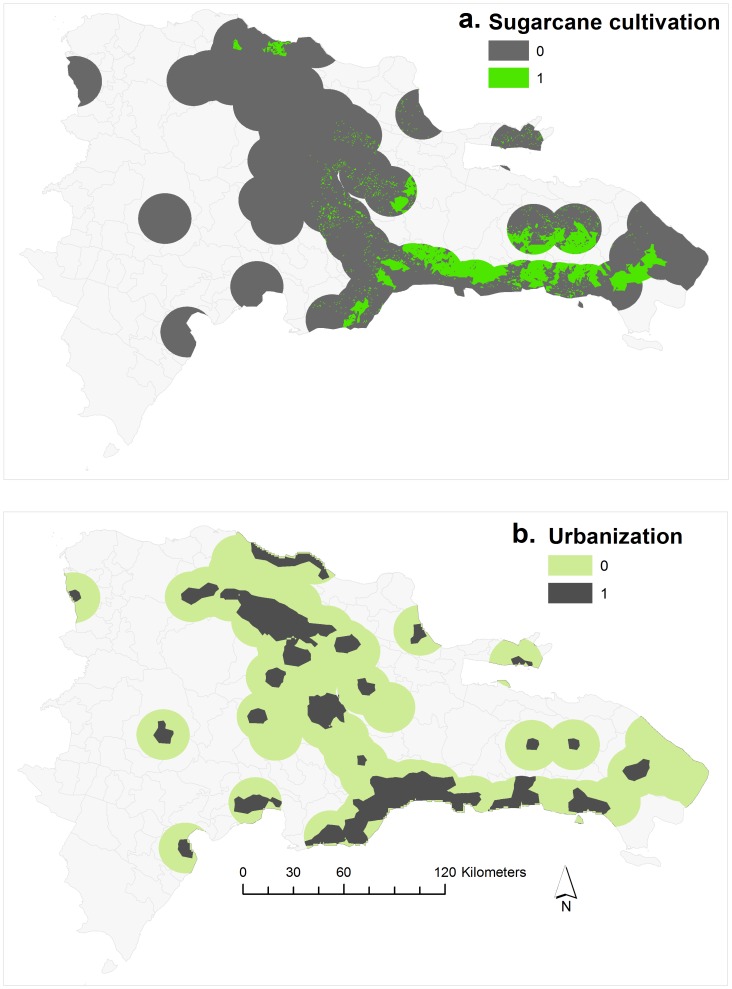
Two of the place-based attributes used in the LDA analysis predicting community membership in the Dominican Republic. Panel a (top) displays sugar cane production and panel (b) displays urbanization. Area shown represents a 15 km radius around mobile phone towers.

**Figure 4:**
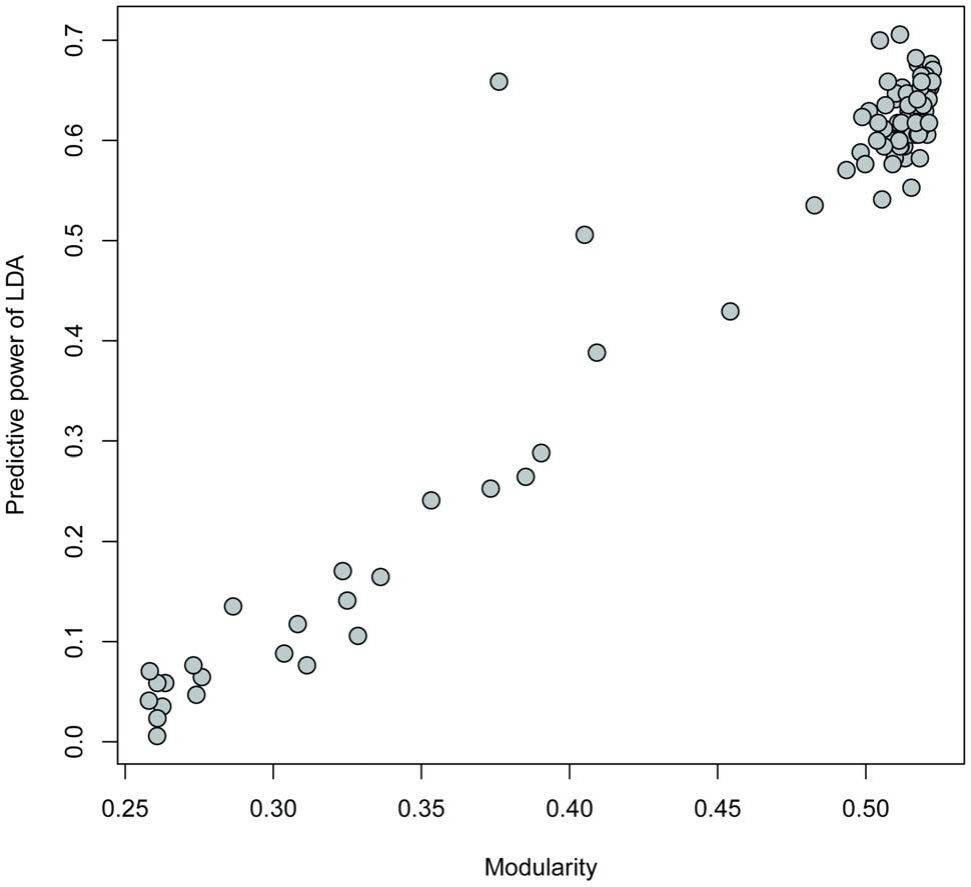
Correlation between predictive power of LDA and modularity score for 100 runs of the modularity algorithm. Modularity values closer to zero represent runs with fewer differences in connectivity between communities than within communities. Predictive power of LDA represents the percentage of towers with community membership correctly predicted.

Towers varied in their place-based attributes (Figure 3, Table 1). For the 100 runs of the LDA, correct predictions of tower community membership ranged from a minimum of 0.5%, corresponding to a modularity score of 0.26, to a maximum of 70.59%, corresponding to a modularity score of 0.51. Indeed, the predictive capability of each LDA model was strongly correlated with the modularity score (Figure 4; Pearson’s linear correlation coefficient r = 0.97). This result demonstrates that runs with higher modularity, presumably closer to the underlying “true” community structure, are better able to be explained by geographic context of towers than runs with low modularity indices.

**Table 1:** Characteristics of place-based attributes used to quantify geographic context.

Place-based attribute	Spatial scale	Range	Mean±SD
Land used for sugar cane production	15 km radius around tower	0–0.54	0.17±0.15
Distance to nearest airport (km)	8 airports in country	1.4–125	20.45±17.96
Urbanization	15 km radius around tower	0–0.96	0.58±0.32
Wealth index	2nd level administrative unit (municipio)	0–0.62	0.45±0.15

In general, the LDA performed well in reconstructing the qualitative features of the communities in the Dominican Republic (Figure 1b). For instance, both the major cities on the island (Santo Domingo and Santiago) were correctly assigned to the appropriate communities by the LDA, and predictions included the distinctive separation between the east and west halves of Santo Domingo. The mobile phone towers that were incorrectly assigned to communities by the LDA tended to be located in outlying areas, such as the towers in the far west of the country near the border with Haiti.

Finally, we assessed the relative importance of our four place-based attributes on community structure in the LDA applied to the run with the highest modularity score. The first, second and third discriminant functions explained 44.1%, 40.3% and 12% of between group variance, respectively (Table 2). Sugar cane land use had the largest coefficients in standardized discriminant functions one and two, followed by urbanization in discriminant function one and the wealth index in discriminant function two. The third discriminant function was dominated by the wealth index. Distance to the nearest airport did not appear to play an important role in the LDA, and was the least important predictor variable in all of the discriminant functions. Qualitatively similar results were observed for other high-ranking runs of the modularity algorithm.

**Table 2:** Output from a linear discriminant analysis for the realization of the modularity algorithm with the highest modularity score.

	Discriminant function			
Predictor	LD1	LD2	LD3	LD4
Sugar cane	7.26	10.97	0.70	1.64
Urbanization	4.27	−3.58	−4.12	−2.47
Rich quintiles	1.61	−3.95	9.26	7.67
Airport distance	0.04	−0.02	−0.04	0.06
**Proportion of trace**	**0.44**	**0.40**	**0.12**	**0.04**

## Discussion

We have demonstrated a close link between community membership and place-based attributes for a large-scale social network of mobile phone communication in the Dominican Republic. This link is evident in the high (>70%) predictive capability of a linear discriminant analysis of community membership based on place-based attributes, and the strongly positive correlation between LDA predictive capability and modularity for 100 separate runs of the modularity algorithm. These results imply that community formation between locations in this mobile phone communication network is related to geographic context, including social structure, wealth distribution, economic production and land use. Consequently, place-based attributes could be used to predict community membership for locations that are not included in the network.

The general principles of homophily [10] and focus constraints [12] have been shown to predict group membership in small-scale social networks. Whether groups of individuals similarly form bonds with other groups of individuals on much larger spatial scales remains an important unaddressed question, because general theories are required to understand the processes underlying community formation in large-scale social networks. In this study, we demonstrate that processes which cause community formation among individuals may also drive community formation in large-scale networks composed of groups of individuals. For example, homophily may explain why communication is higher between towers in the wealthy western half of Santo Domingo than between western towers and towers in the less wealthy, eastern half of the city. One group of towers which was not correctly assigned community membership by the LDA were the towers in the west of the country near the border with Haiti, which the modularity algorithm determined were in the same community as towers in Santo Domingo. This discrepancy could reflect a focus constraint between tower locations not captured by our set of place-based attributes, such as wealthy Dominican urbanites hiring Haitian laborers for housekeeping services.

The amount of land used for sugar cane cultivation was the most important predictor of community membership. Although sugar cane production is no longer a dominant economic activity in the Dominican Republic, the importance of sugar cane in determining community membership likely reflects deep, underlying differences in social structure between regions. These differences may be driven by the long-term social effects of class divisions arising from the mode of sugar cane cultivation in large plantations, compared to the country’s other main agricultural crops, which are produced by more egalitarian collectives of small farmers [20]. Here we have demonstrated that community structure can be predicted accurately using a choice of just four relevant place-based attributes to quantify geographic context. These represent only a fraction of the potential additional attributes that could be used to describe the location of nodes in large-scale networks, and the addition of a broader range of place-based attributes, including ethnicity, gender or health infrastructure, may further aid explanation of the underlying community patterns, and should be a focus of future research.

One challenge for future research is to incorporate both space and place-based attributes into quantitative models for community membership. Several previous studies have demonstrated that space plays an important role in determining patterns in large-scale social networks [8], [14]–[15]. Expert (2011) has presented a method for modularity which removes the effect of space from community membership in the modularity algorithm [15]. A disadvantage of this approach is that if the goal of analysis is to predict community membership, and space drives the pattern of modularity, removing space using the modularity algorithm eliminates an important element leading to community formation. In contrast, the method we present, using an LDA with place-based attributes can account for spatial autocorrelation due to the spatial distribution of predictor variables. Our method is unable to account for patterns in modularity which are the result of space alone, for example, if nearby towers are more likely to communicate, regardless of any shared social or economic attributes. However, results from a k-means clustering analysis classifying towers into 13 communities based solely on pairwise distance between towers, suggest that spatial proximity alone does not capture many features of the observed community structure (Figure 2). A method of analysis which simultaneously quantifies the effect of space and place-based attributes on community membership is an important goal for future research.

There are several limitations to the dataset used. First, although per capita mobile phone ownership in the Dominican Republic is relatively high [21], mobile phone use may be biased towards wealthier and working age individuals. Despite these heterogeneities in mobile phone ownership, the most extensive study of mobile phone ownership to date has found that every region, income and demographic bracket is represented in mobile phone datasets [22]. Additionally, the mobile phone network used comprised only 5% of market share in the Dominican Republic, meaning results may have limited generality if mobile phone coverage or call flow is very different for other in-country mobile phone companies. Finally, mobile phone towers are placed non-randomly, usually in places with high population density [23], and geographic context of towers reflects this non-random placement; however, placement alone seems unlikely to explain many of the striking patterns we observed in the data, such as the division between the east and west halves of Santo Domingo. Despite these caveats, our findings show clear patterns that match with existing geographical knowledge on the Dominican Republic, and also demonstrate the application of a method for quantifying the influence of geographic context on modularity.

Because mobile phone communication networks are related to economic activity [17], friendship [1], and human mobility [16]–[19], the ability to predict patterns of communication across regions has major implications for a range of fields, from epidemiology to political science. Our results suggest that place-based attributes related to social, economic and ecological context can predict community membership in mobile phone communication networks. Consequently, the potential to extrapolate community membership across wide regions not covered by mobile phone towers exists.

## Methods

We used a dataset composed of the symmetrized number of calls between 177 mobile phone towers in the Dominican Republic. These data represent a single mobile phone company with 5% of Dominican mobile phone market share. The 57 million mobile phone communications in this dataset occurred between June 2007 and May 2008. For each call, the tower used by the phone initiating the call (the “origin” tower) and the tower used by the telephone receiving the call (the “destination” tower) were recorded. Over the entire study period, the mean call volume originating from each tower was 323655 (standard deviation 240468). For each pair of towers A and B, the number of calls originating from A calling phones serviced by B was known, as well as the number of calls originating from B to phones in A. In this dataset, these two values were not necessarily equal. Following previous analyses of country-wide mobile phone communication networks (4), we symmetrized calls going between a pair of towers by setting the number of calls from A going to B and the number of calls from B going to A equal to the lesser of the two values. Symmetrizing in this manner did not significantly affect the data; the mean discrepancy between the two values for pairs of towers was 3%. The total number of nodes in the network is 177 (the number of mobile phone towers) and the number of links between towers is 57286839. We excluded towers identified in the dataset as repeaters from analysis, because these towers are used to boost signals from other towers, rather than servicing a particular location. This network of towers covered much of the population of the Dominican Republic, and serviced a wide range of urbanization, wealth and land use (Figure 2, Table 1).

We analyzed existing data from the mobile phone company, which originally had been collected for billing purposes, not for the purposes of this study. This mobile phone network was rendered anonymous by the mobile phone company before we accessed the data, making it impossible to identify individual mobile phone users. Additionally, the data were aggregated as the sum of calls from one tower to another, making it impossible to extract information on individuals from the dataset. The anonymity and aggregation of the data are strong safeguards maintaining the privacy of individual mobile phone users in the dataset.

We quantified community structure in the network using modularity maximization with a simulated annealing algorithm [25]. Previous research has shown that modularity maximization is a particularly effective approach for detecting communities in networks [7] and modularity maximization has been successfully applied to several mobile phone communication networks [6], [15]. This algorithm determines the number of communities and node membership in communities by maximizing the difference in calls within a community and calls between communities. We used a weighted version of modularity, with a null model in which edges are placed at random, with the constraint that the degree of a vertex in the null model is equal to the degree of that same vertex in the real network [25]. The output of this algorithm is community membership of each node and modularity, an index representing the degree of clustering from 0 (modularity not significantly different than edges distributed at random) to 1. Determining the global maximum of the modularity function requires calculating the modularity score for all possible partitions of the network into communities. In fact, optimizing modularity is an NP-hard problem, meaning that optimization algorithms can only approximate the optimal modularity score [24]–[26]. Furthermore, there may be many local optima in the modularity surface, arising from different partitions of the network into communities. To address this concern, we conducted 100 separate runs of the modularity optimization algorithm. For each run, we extracted the optimum modularity score and the community membership of each mobile phone tower.

We analyzed the process determining community formation in the call network by relating community membership to place-based attributes using a linear discriminant analysis (LDA). We selected four variables to use as predictors of community membership in the LDA: area occupied by urbanized land, area used for sugar cane production, distance to nearest airport and income at the second level administrative unit. Each of these variables represents an economic, social or ecological element of each tower’s landscape. Urbanization is central to many social processes in Latin America [27]. We chose land cover used for sugar cane production as a place-based attribute because social networks in regions of the country with sugar cane plantations have fundamental differences, including higher social stratification and economic inequality, relative to social networks in regions with other forms of agriculture [20]. Both urbanization and sugar cane land cover were quantified from satellite imagery datasets [28]–[30] using a 15 km radius around each tower (Figure 2), an area chosen as an appropriate scale to represent the maximum area that a signal from an individual mobile phone tower could occupy. We quantified distance from each tower to the nearest of 8 airports, because we anticipated the proximity to airports would reflect economic conditions related to international tourism, which is a major source of income for the Dominican Republic [31]. Finally, we measured income as the proportion of people in each second-level administrative unit belonging to the highest quintile of wealth, as measured by the Wealth Index developed by MEASURE DHS (www.measuredhs.com). Although the MEASURE DHS data contains many other predictor variables related to socioeconomic status, such as motorcycle ownership, poverty rates and presence of indoor plumbing, multicollinearity between socioeconomic variables limited the number of predictor variables we could include in the model. Consequently, we chose to use the Wealth Index, because this measure of wealth correlates much more strongly with socioeconomic status than most other measures, as measures such as self-reported income can be highly inaccurate, can change over the course of a year, and may not include all sources of income [21]. Upon determining the proportion belonging to the highest quintile of wealth in each second-level administrative unit (“municipio”), we associated that proportion with every tower in each municipio. We chose to aggregate this proportion over municipios rather than aggregate over each tower because the MEASURE DHS data is weighted to be aggregated to the first level administrative unit. Our LDA analysis had two objectives: first, to quantify the predictive power of the LDA for community membership, and second, to analyze the relative explanatory power of our four predictor variables. We determined whether our place-based attributes were able to correctly predict community membership of towers by applying an LDA with leave-one-out cross-validation to all 100 realizations of the modularity algorithm. This approach provides a value of predictive capability linked to each modularity realization. Calculating a range of predictive capabilities linked to various levels of modularity has the benefit of enabling us to link the pattern described by the modularity algorithm with the process quantified by the LDA. If the LDA is accurately reflecting the processes which drive community membership, we would expect the predictive capability of the LDA to increase as the modularity index increases.

To evaluate which predictor variables were most important for determining community membership, we conducted an LDA using the realization of the modularity algorithm with the top modularity score. Repeating the analysis for other realizations revealed qualitatively similar results. Because the goal of analyzing the single LDA for the top modularity realization was explanation, rather than prediction, we included the full dataset in this analysis. To assess the relative importance of predictor variables, we examined the proportion of between group variance explained by each of the four discriminant functions and the coefficients of the standardized discriminant function.

## Supporting Information

Figure S1 The simulated annealing runs with the 20 highest modularity scores (Q >.52, out of 100 total runs) were used to determine core communities. Core communities were defined as communities with at least 3 towers that appeared in every run, where each core tower was a member of the same community for all 20 runs. Towers that were not assigned to the same community throughout all 20 runs were termed non-core towers, and are represented as green open circles, while core communities are represented with filled circles, with colors corresponding to the communities in Figure 1.
(TIF)

Video S1 This brief video presents our methods and results in language appropriate for a general audience.
(MOV)
